# Longitudinal circulating tumor DNA profiling in patients with advanced endometrial cancer using an off‐the‐shelf targeted NGS panel

**DOI:** 10.1002/1878-0261.70246

**Published:** 2026-04-28

**Authors:** Carlos Casas‐Arozamena, Karin Teien Lande, Eva Diaz, Ana Vilar, Juan Cueva, Efigenia Arias, Victoria Sampayo, Alicia Abalo, Nerea González, Eva Colás, Antonio Gil‐Moreno, Miguel Abal, Gema Moreno‐Bueno, Therese Sørlie, Kristina Lindemann, Laura Muinelo‐Romay

**Affiliations:** ^1^ Translational Medical Oncology Group (Oncomet), Health Research Institute of Santiago de Compostela (IDIS) University Hospital of Santiago de Compostela (SERGAS) Santiago de Compostela Spain; ^2^ Section for Gynecological Oncology, Department of Surgical Oncology Oslo University Hospital Oslo Norway; ^3^ Department of Cancer Genetics, Institute for Cancer Research Oslo University Hospital Oslo Norway; ^4^ MD Anderson Cancer Center Foundation Madrid Spain; ^5^ Department of Gynecology University Hospital of Santiago de Compostela (SERGAS) Santiago de Compostela Spain; ^6^ Department of Gynecologic Oncology, Biomedical Research Group in Gynecology, Vall Hebron Research Institute (VHIR) Universitat Autónoma de Barcelona Barcelona Spain; ^7^ Centro de Investigación Biomédica en Red de Cáncer (CIBERONC) Madrid Spain; ^8^ Instituto de Investigaciones Biomedicas “Sols‐Morreale” CSIC‐UAM Madrid Spain; ^9^ Translational Cancer Research Group, Area 3 Cancer Health Research Institute Ramon y Cajal (IRYCS) Madrid Spain; ^10^ Institute of Clinical Medicine, Faculty of Medicine University of Oslo Norway; ^11^ University of Santiago de Compostela (USC) Santiago de Compostela Spain

**Keywords:** ctDNA, disease evolution, endometrial cancer, liquid biopsy, tumor kinetics

## Abstract

Intratumor heterogeneity presents a major challenge in precision oncology for endometrial cancer (EC). Circulating tumor DNA (ctDNA) offers a minimally invasive method to monitor tumor evolution and therapeutic resistance. In this retrospective study, we evaluated a tumor‐agnostic NGS panel to detect and track ctDNA in 18 EC patients and directly compared its performance with a tumor‐informed ddPCR approach. ctDNA was detected by NGS in over 60% of plasma samples, while ddPCR showed higher positivity rates in paired samples (71.9% vs 62.5%), with overall concordance of 65.7% and fair agreement (Cohen's kappa = 0.23). The cfDNA‐NGS panel identified a broad spectrum of alterations, including relapse‐specific mutations indicative of clonal evolution, but showed lower sensitivity for low‐frequency variants compared with ddPCR. Discordant cases, including false‐negative results in both approaches, highlight the impact of assay sensitivity, target selection, and biological factors on ctDNA detection. ctDNA dynamics correlated with disease progression and treatment response, although detection was limited in cases with brain metastases. These findings support the utility of tumor‐agnostic ctDNA monitoring in advanced EC and highlight the importance of assay quality and careful interpretation to address limitations such as clonal hematopoiesis and technical sensitivity.

## Graphical abstract

Intratumour heterogeneity complicates precision management of advanced endometrial cancer. Circulating tumor DNA (ctDNA) offers a minimally invasive strategy to capture tumor evolution and therapeutic resistance. Here, we compare tumor‐agnostic NGS with tumor‐informed ddPCR, outlining their relative sensitivity, concordance, and clinical implications. We discuss how assay design, clonal evolution, and biological context shape ctDNA detection and interpretation in clinical practice.

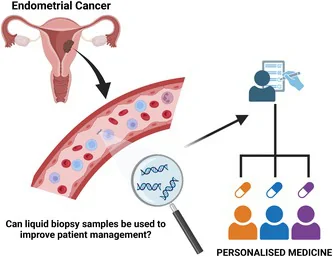

**Abbreviations**
AF: allelic frequency
cfDNA: circulating free DNA
CH: clonal hematopoiesis
CNA: copy number alteration
ctDNA: circulating tumor DNA
ddPCR: droplet digital PCR
dMMR: mismatch repair‐deficient
EC: Endometrial Cancer
EEC: endometrioid endometrial carcinomas
FIGO: International Federation of Gynaecology and Obstetrics
IHC: immunohistochemistry
LoD: Limit of detection
MSI‐H: microsatellite instability high
NEEC: nonendometrioid endometrial carcinomas
NGS: next‐generation sequencing
PD: progressing disease / relapse
SNP: single nucleotide polymorphism
SNV: single nucleotide variants
t‐NGS: targeted NGS
UA: uterine aspirates
VAF: variant Allelic frequency

## 1 Introduction

Precision oncology aims to advance cancer treatment toward a tailored approach to improve diagnosis, prognostication, and therapy selection. Intratumor heterogeneity is one of the main obstacles that precision oncology is meant to tackle, as variability within tumors could lead to differences in treatment responses and often subsequent development of resistance to targeted therapies [1]. This may be especially important for patients with favorable prognostic features at diagnosis, whose disease can still progress aggressively and respond poorly to treatments.

Patients with endometrial cancer (EC) often present significant intratumor heterogeneity, and it has been associated with more aggressive tumors [2, 3]. Although EC often is associated with a good clinical outcome, a significant proportion of patients (20–30%) are diagnosed with advanced disease, with the 5‐year survival rate dropping to barely 20% [4]. Traditional treatment strategies for EC have primarily relied on a combination of surgery, radiation, and chemotherapy, guided by tumor histology and stage. Of note, immunotherapy has become an important treatment option for EC. Initial studies demonstrated that patients with mismatch repair‐deficient (dMMR) or microsatellite instability‐high (MSI‐H) tumors benefit from single‐agent immunotherapy in the recurrent setting. Since then, its use has expanded, with indications in advanced but also recurrent setting for patients with dMMR and non‐MMR tumors [5]. Also, HER2 targeting treatment strategies and as well as other targeted therapeutic avenues are increasingly explored. Despite these recent advances in targeted and immunotherapy, further improvements in patient response and outcomes will require a deeper understanding of the genomic landscape, particularly throughout disease progression and at relapse [6, 7, 8].

Previous studies have shown that the EC genomic landscape is dynamic and substantial changes can occur through the course of the disease [9, 10]. Liquid biopsy analyses enable not only the dynamic characterization of the disease, using minimally invasive samples, but also capture tumor heterogeneity [11, 12, 13]. Various technologies have been employed to study circulating tumor DNA (ctDNA) in plasma from EC patients, including tumor‐informed strategies using targeted ddPCR (droplet digital PCR) or qPCR [14, 15, 16, 17, 18, 19, 20], as well as tumor‐agnostic methods using NGS (next‐generation sequencing) platforms [21, 22, 23, 24, 25, 26]. Although research of ctDNA kinetics in EC is still limited, previous studies performed by us and others indicate that circulating cell‐free DNA (cfDNA) and ctDNA analyses represent a valuable strategy to detect early relapses, monitor treatment responses, and identify resistance mechanisms [14, 21, 26]. These studies have demonstrated the feasibility of identifying ctDNA in patients with both localized and advanced endometrial tumors, with higher ctDNA levels correlating with more aggressive disease phenotypes. Recently, ctDNA analysis for patients in the Duo‐E trial [27, 28] not only confirmed the prognostic significance of ctDNA in a clinical trial setting but also demonstrated the feasibility of ctDNA monitoring of a treatment effect. Even though tumor‐informed methods usually offer the high analytical sensitivity necessary for ctDNA analysis, they are limited to detecting only a small number of hotspot mutations. In contrast, clinical decision‐making increasingly requires broader mutation profiling, but often to the costs of a reduced level of detection. Unlike tumor‐informed approaches, off‐the‐shelf cfDNA panels do not require prior tumor profiling, allowing immediate and repeatable ctDNA testing while enabling detection of newly emerging clones during disease progression. We therefore hypothesize that an off‐the‐shelf cfDNA‐NGS panel can provide a practical and clinically scalable strategy to detect relevant genomic heterogeneity and clonal evolution over time.

Despite increasing use of ctDNA in EC, the optimal strategy for routine monitoring remains unclear. To address this gap, and building on our previously published cohort of EC patients monitored with a tumor‐informed ddPCR approach [14], we evaluated whether this NGS approach, successfully applied in baseline samples from endometrial tumors [26], could provide additional clinical value. In a subset of 18 patients, we assessed the panel's ability to monitor disease evolution and compared its performance with that of the tumor‐informed ddPCR assay. Our goal was to determine the potential of this broader, tumor‐agnostic approach for routine implementation in the management of EC patients.

## 2 Material and methods

### 2.1 Sample inclusion and processing

A total of 18 patients with EC were selected from a retrospective cohort between January 2018 and June 2023 from the Gynaecology Department of Vall d'Hebron University Hospital (Barcelona, Spain), the MD Anderson Cancer Center (Madrid, Spain), and the University Clinical Hospital of Santiago de Compostela (Santiago de Compostela, Spain). Patients were included in the study if the following criteria were met: (a) patients were diagnosed with endometrial carcinoma of any histology (excluding sarcomas or carcinosarcomas); (b) patients were over 18 years old; (c) patients signed informed consent; (d) patients were not undergoing anti‐tumor treatment at the time of inclusion; (e) patients did not have any other malignancy within the last 5 years before the sample collection; (f) data on the patients' mutational profiles were known (Table S1); (g) data about ctDNA by means of ddPCR‐based characterization were available. The study was approved by the corresponding Research Ethics Committees (Galician Research Ethics Committee—reference number 2017/530 and 2022/029, Vall d'Hebron Research Ethics Committee—reference number PRAMI276‐2018) and conducted in accordance with the guidelines for Good Clinical Practice and the Declaration of Helsinki. All patients provided written informed consent before enrolment.

Peripheral blood samples (*n* = 32) from the 18 patients were collected in CellSave tubes (Silicon Biosystems Inc, Huntington Valley, USA) when patients had confirmed active tumor disease, either at baseline before treatment (*n* = 12), at first relapse (PD) (*n* = 10), or at sequential progressions (*n* = 10). Uterine aspirates (UAs) from all patients were collected at the time of surgery and processed for the DNA characterization by targeted sequencing as previously published [14]. The UA and the baseline plasma samples were collected at the same time, during surgical resection in all patients. A summary of clinicopathological features of this cohort and the time points for ctDNA assessment are listed in Table 1. Plasma was isolated following a two‐step centrifugation protocol within 8 h after blood collection. The isolated plasma was stored at −80°C until further processing.

**Table 1:** Summary of the most relevant clinicopathological features in the cohort. EEC, Endometrioid endometrial cancer; HCN, high–copy number; MSI, Microsatellite instable; NEEC, Nonendometrioid endometrial cancer; NSMP, nonspecific molecular profile.

ID	Histology	Grade	FIGO	Myometrial infiltration	LVSI	Molecular classification	Risk of recurrence (ESGO 2023 [31])	Relapse	Sample collection
ID01	EEC	G1	II	≥50%	No	NSMP	High‐Intermediate	Yes	Relapse
ID02	NEEC (Serous)	G3	II	<50%	No	HCN	High	Yes	Relapse
ID03	NEEC (Serous)	G3	I	≥50%	No	HCN	High	Yes	Baseline and relapse
ID04	EEC	G2	I	<50%	No	HCN	Low	No	Baseline
ID05	EEC	G2	II	≥50%	No	MSI	High‐Intermediate	Yes	1st and 2nd relapse
ID06	EEC	G3	I	≥50%	Yes	HCN	Intermediate	Yes	During treatment after 1st relapse, 2nd and 3rd relapse
ID07	EEC	G3	I	≥50%	Yes	MSI	High‐Intermediate	Yes	Baseline, before treatment after 1st relapse and at 2nd relapse
ID08	EEC	G3	I	≥50%	No	MSI	High‐Intermediate	No	Baseline and 4th relapse
ID09	EEC	G2	I	≥50%	No	NSMP	Intermediate	Yes	Baseline
ID10	EEC	G2	I	<50%	No	MSI	Low	No	Baseline
ID11	EEC	G3	III	<50%	No	MSI	High	Yes	Baseline
ID12	NEEC (Serous)	G3	IV	≥50%	Yes	HCN	Advanced	Yes	Baseline and before death
ID13	NEEC (Serous)	G3	I	<50%	No	NSMP	Intermediate	No	Baseline
ID14	NEEC (Serous)	G3	I	<50%	No	HCN	Intermediate	Yes	1st, 2nd and 3rd relapse
ID15	NEEC (Serous)	G3	IV	≥50%	No	HCN	Advanced	Yes	Baseline and relapse
ID16	NEEC (Serous)	G3	III	≥50%	Yes	MSI	High	Yes	Baseline, 1st and 2nd relapse
ID17	EEC	G2	I	≥50%	No	MSI	Intermediate	Yes	Baseline and after relapse before treatment
ID18	EEC	G1	I	<50%	No	NSMP	Low	No	Baseline

### 2.2 Nucleic acid isolation

DNA from plasma samples was extracted with the QIAamp DNA Circulating Nucleic Acid Kit (Qiagen, Hilden, Germany), according to the manufacturer's instructions. All cfDNA extractions were performed using 3–5 mL of plasma, and the concentration was normalized based on the extraction volume and the elution volume. Samples were stored for a maximum of 10 months before the cfDNA was isolated. DNA and RNA from the UA were obtained using the RecoverAll™ Total Nucleic Acid Isolation Kit (Thermo Fisher Scientific, Waltham, MA, USA) following the manufacturer's conditions. All DNA samples were quantified using the Qubit Fluorometer (Thermo Fisher Scientific) and stored at −20°C until use.

### 2.3 Sequencing analyses

Isolated cfDNA was quantified using Qubit, and integrity was measured using the TapeStation cell‐free DNA screen tapes (Agilent Technologies, Santa Clara, CA, USA). After quality control, the cfDNA was sequenced using the Oncomine™ Pan‐Cancer cell‐free assay (Thermo Fisher Scientific) according to the manufacturer instructions and as previously described [26]. The panel covers 52 genes, including SNPs, gene fusions, CNAs, and tumor suppressor genes. Briefly, manually prepared targeted sequencing libraries were generated using the Oncomine™ Pan‐Cancer Cell‐Free Assay (Thermo Fisher Scientific). The total input of cell‐free tumor nucleic acid ranged from 4.63 to 58.24 ng (median = 20.23 ng). After library preparation, the quality was screened using the Tape Station D1000 and were quantified using the Ion Library TaqMan quantification kit. Subsequently, 100 mm of the library was loaded onto Ion 540 chips via Ion Chef and sequenced using the Ion GeneStudio™ S5 system (Thermo Fisher Scientific). Data output was processed using Ion Reporter (IR) software (Thermo Fisher Scientific) with the ‘Oncomine TagSeq Pan‐Cancer Liquid Biopsy w2.1’ workflow using the standard parameters. A summary of the coverage and other metrics can be found on Table S3. In brief, the average number of mapped reads was of 17 M [10 M–26 M], with an average of 93% on target and uniformity of base coverage of 97%.

Single nucleotide variants (SNVs) with an allele frequency (AF) of approximately 50% (±7%) or considered as benign according to Varsome (www.varsome.com) were excluded. For each detected SNV and InDel, the quality control limit of detection (LoD) was used as a threshold for (technical) significance of the mutations. The LoD was calculated per locus. Mutation (SNV or INDEL) AF above this LoD (AF > LoD) was considered significant and the respective sample labeled as ctDNA positive. The mutation molecular coverage was above 8 for all significant mutations and hence well above the default threshold of 3. Samples with no mutational hits were considered as noninformative. Two quality control LoD values are given by IR: the median value across all targets and the value for the 80th percentile targets. A mean of these two was calculated and used as the LoD measure for the noninformative samples.

DNA and RNA extracted from UAs were sequenced using the Oncomine Comprehensive Panel v3 (Thermo Fisher Scientific) according to previously published protocols [14]. Briefly, 10 ng of DNA and cDNA from each UA were used for library preparation via multiplex PCR on an AB2720 Thermal Cycler (Life Technologies, Carlsbad, CA, USA), followed by primer digestion with FuPa reagent and amplicon ligation using Ion P1 Adapter and Ion Xpress Barcode X (Thermo Fisher Scientific). Libraries were purified, quantified (Ion Library TaqMan Kit, ViiA 7 system), and diluted to match control standards. Template preparation and sequencing were performed on the Ion S5 XL system with 540 chips (Thermo Fisher Scientific). Duplicates in 10% of samples showed consistent results. Bioinformatic analysis included alignment to Hg19 and variant calling via Torrent Suite Software v.15.1 (Life Technologies). Low‐quality variants (Phred <100) were excluded, and functional predictions were assessed with Alamut Visual Plus. Variants with a minor allele frequency >0.05 (dbSNP) were classified as SNPs and excluded. Genetic susceptibility variants were validated using an alternative sequencing method.

### 2.4 Tailored ddPCR analyses

ddPCR assays were run as previously described [14]. When feasible, all mutations found on the UA were screened using the ddPCR approach (Table S1). In short, reactions used 30 ng cfDNA when available, with a minimum of 10 ng per assay. The median cfDNA input for ddPCR was of 25 ng. PCR was performed using Bio‐Rad's ddPCR Supermix and samples were partitioned into ~50 000 droplets across triplicates using an automated droplet generator (Bio‐Rad Laboratories, Hercules, CA, USA). Emulsified PCR reactions were run on a C1000 Touch™ thermal cycler (Bio‐Rad Laboratories), and results were analyzed with a Bio‐Rad QX‐200 droplet reader (Bio‐Rad Laboratories) and QuantaSoft v1.7.4 software (Bio‐Rad Laboratories). Two independent molecular biologists manually reviewed results, requiring ≥30 000 positive droplets for validity and at least five single FAM‐ or HEX‐positive droplets with ≤2 positive events in WT controls to confirm mutations. ddPCR was repeated if contamination was found in negative controls or if >2 events appeared in WT controls. The limit of blank and LoD were set at 0.04% [0.01–0.05%] and 0.1% [0.05–0.2%], respectively. To ensure the functionality of the probes, the UA was used as an added positive control to validate the results.

### 2.5 Statistical analyses

Statistical differences in nonparametric variables were evaluated using Wilcoxon's rank‐sum and matched‐pairs signed rank tests. Correlation analyses employed Spearman's rank correlation coefficient. Concordance between ctDNA methods was assessed using Cohen's kappa statistic, with values interpreted as slight [0.01–0.20], fair [0.21–0.40], moderate [0.41–0.60], substantial [0.61–0.80], and almost perfect agreement [0.81–1.00]. Agreement between continuous measurements from two methods was further evaluated using Bland–Altman analysis. A *P*‐value <0.05 was set as the level of statistical significance. Data visualization was performed using the ggplot2 package [29]. Analyses were conducted using R (version 4.2.3 [30]) on Windows 10 ×64 (build 22 631).

## 3 Results

### 3.1 Cohort characteristics

The cohort consisted of patients with endometrioid (EEC) or non‐endometrioid (NEEC) adenocarcinomas, varying grade of differentiations, and FIGO stages I to IV, classified according to the latest ESMO/ESGO consensus guidelines [31]. Notably, as illustrated in Table 1, the cohort primarily consisted of high‐risk patients who experienced relapse, representing 72% (13/18) of the total cohort. The genetic profile of each tumor has been previously published as part of a study of a larger cohort using the Oncomine Comprehensive Panel v3 on the uterine aspirate (UA) from the patients [14]. The most commonly altered genes within this subset of patients were *PIK3CA* (72.22%), *TP53* (33.33%), *ARID1A* (33.33%), *PTEN* (33.33%), and *FGFR2* (22.22%) (Table S1).

### 3.2 ctDNA mutational profile

**Fig. 1:**
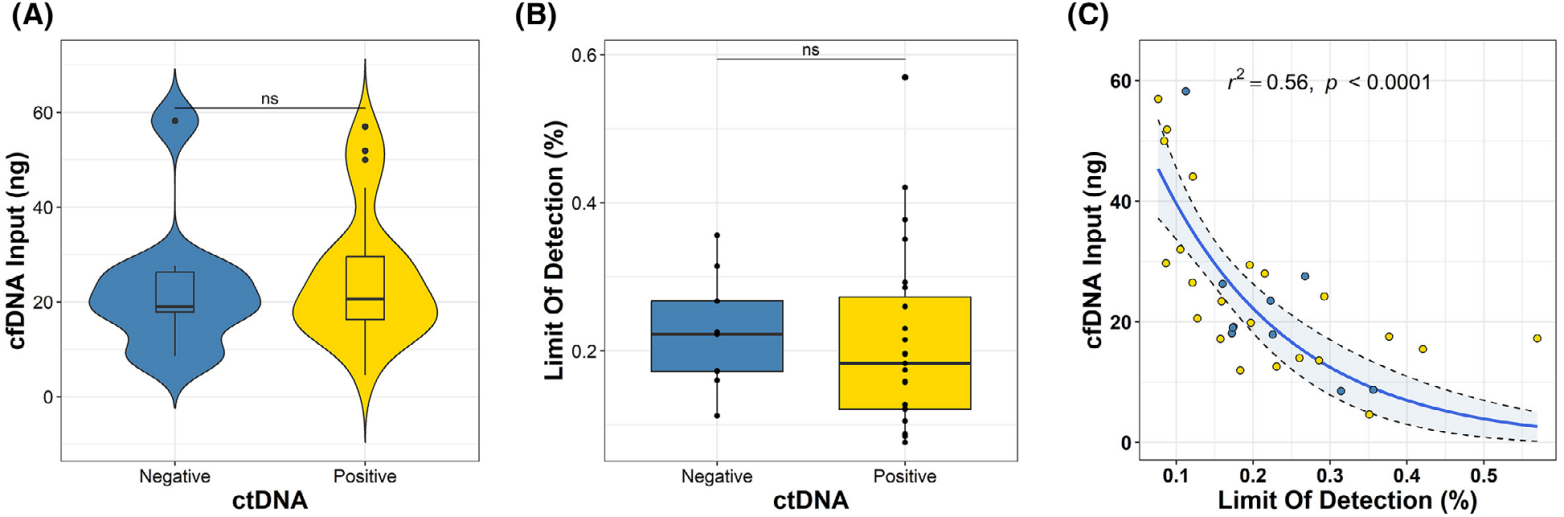
Summary of the quality metrics for the NGS analyses of the cfDNA. (A) Violin plot illustrating the amount of DNA input (ng) for the circulating tumor DNA (ctDNA) negative and ctDNA‐positive patients. Errors bars indicate SD. Wilcoxon's rank‐sum test *P* > 0.05. (B) Box plot illustrating limit of detection (LoD) (%) for ctDNA‐negative and ctDNA‐positive patients, separately. Errors bars indicate SD. Wilcoxon's rank‐sum test *P* > 0.05. (C) Scatter plot illustrating the correlation between cfDNA input (ng) and the limit of detection (LoD, %), with a fitted nonlinear exponential regression curve (*r* ^2^ = 0.56, *P* < 0.0001). and 95% confidence interval (dotted lines).

All 32 cfDNA samples from the 18 patients underwent NGS analysis with the Oncomine™ Pan‐Cancer Cell‐Free Assay. Samples were considered ctDNA informative (ctDNA positive) if at least one pathogenic alteration was found over the limit of detection (LoD). cfDNA input varied from 4.63 to 58.24 ng with a median of 20.23 ng. No statistically significant differences were observed between patients with detectable and undetectable ctDNA levels (Fig. 1A, Wilcoxon's rank‐sum test *P* > 0.05) in terms of DNA input. The LoD was relatively low (median 0.19%, range 0.076% to 0.56%), without any statistically significant association with the presence of ctDNA (Fig. 1B). As expected, the LoD was exponentially dependent on the cfDNA input (*r* ^2^ ~ 0.6, *P*‐value <0.001, Fig. 1C), supporting the convenience to include 30–40 ng of cfDNA to reach low LoD (~0.05–0.1%).

**Fig. 2:**
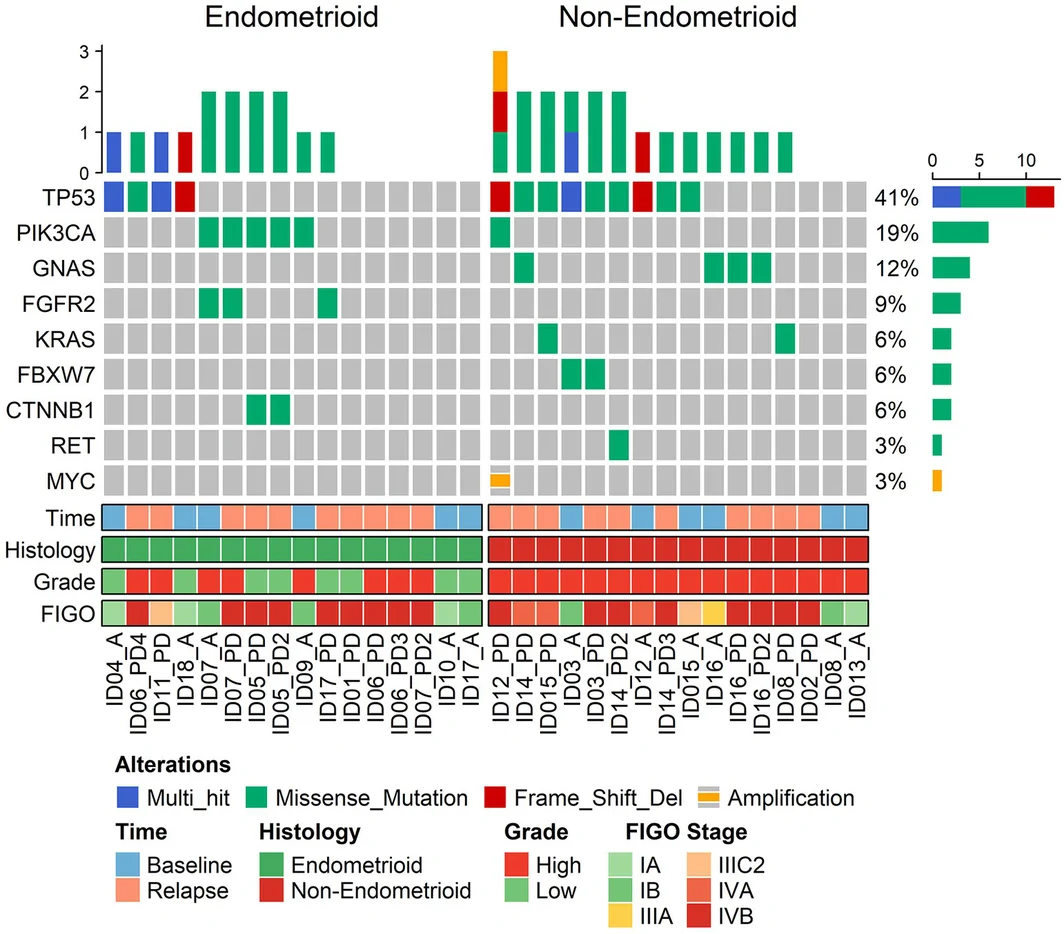
Graphical representation of the identified mutations in the cohort. Heatmap illustrating the identified mutations across all samples according to the time of collection. Columns represent patients whereas rows represent the most frequently altered genes. The right annotation showcases the prevalence and number of events for each mutation in the entire cohort. Top annotation refers to the number of alterations found in each patient. Bottom annotation summarizes the type of alteration, time of collection (at first diagnosis or relapse), and clinicopathological features (histology, grade, and FIGO (International Federation of Gynaecology and Obstetrics) stage).

In terms of sensitivity, 20/32 (62.50%) of the samples showed pathogenic alterations, with slightly higher rates in samples collected at the time of disease relapse 13/20 (65%) compared to baseline 7/12 (58.33%). A detailed summary of these alterations is provided in Table S2. The most frequently altered genes included *TP53* (40.62%), *PIK3CA* (18.75%), *GNAS* (12.50%), *FGFR2* (9.38%), *KRAS* (6.25%), *FBXW7* (6.25%), *CTNNB*1 (6.25%), *RET* (3.13%), and *MYC* (3.13%) (Fig. 2). Most of the alterations were missense mutations (81.08%), a few were frameshift deletions (16.22%), and only one copy number alteration was identified within the cohort (2.70%). Three patients presented multi‐hit alterations on the same gene, *TP53*, potentially indicative of intratumor heterogeneity.

### 3.3 Identification of variants associated with clonal hematopoiesis

One of the main challenges for plasma NGS analyses is discerning DNA of tumor origin and DNA derived from clonal hematopoiesis (CH) [32]. CH is heavily linked to age and the risk of EC increases with age. Therefore, the impact of CH should be taken into consideration during the analyses of these, often, elderly patients. Since no matching leukocytes were available for testing, the following approach was taken.

In our cohort, 18 out of 32 (56.25%) samples presented at least one alteration on a gene related to CH (*TP53*, *GNAS* and *KRAS*) [33]. The variant allelic frequency (VAF) of these genes presented a median of 0.97% (0.1–67.89%). As the prevalence of *GNAS* mutations is relatively low in EC (<1%), they were labeled as highly likely to be related to CH and the corresponding samples were considered ctDNA negative, if they only showed an alteration in this gene. However, since *TP53* and *KRAS* are highly relevant genes in the genomic landscape of EC, samples with *TP53* or *KRAS* mutations were considered ctDNA positive, including one case with mutations in *TP53* as well as in *GNAS*. Therefore, within our cohort, 3 out of 32 (9.37%) samples were considered as highly likely of presenting CH and those samples were considered as noninformative for all analyses.

### 3.4 Concordance between the uterine aspirate and the cfDNA by NGS

To assess the robustness and reliability of the assay, we compared the results obtained from the cfDNA using the t‐NGS panel with the sequencing results from the UAs obtained from the same patients [14]. The UAs were collected at the time of surgical resection and were subjected to a different t‐NGS panel, the Oncomine™ Comprehensive Panel v3. This panel includes 161 genes: 130 genes for hotspot or full gene coverage, 43 for focal CNV, and 51 for fusion drivers. From these, the following are covered by the Oncomine™ Pan‐Cancer Cell‐Free Assay: 43 genes (31.6%) for hotspot mutations, 12 genes (27.9%) for copy number, and 12 genes (23.5%) for fusion. Cases were considered concordant if the same mutation was found in both samples. In samples with multiple alterations, at least one mutation needed to be the same to be considered concordant.

Concordance was defined as the presence of at least one identical SNV or CNA in both the UA and cfDNA samples. No fusions were detected. Because the two panels differ in gene coverage, concordance analysis was restricted to 17 patients in whom at least one UA alteration was also covered by the cfDNA panel (Table S1). Among these, 10 of 17 patients (58.8%) showed at least one concordant alteration. Overall, 13 of 30 samples (43.3%) were concordant. Notably, concordance was higher in the relapse setting (10 of 18, 55.6%) than at baseline (3 of 11, 27.3%). Only SNVs were found concordant between the UA and plasma samples. Since only two patients presented CNAs, the concordance ratios could not be robustly assessed. Median depth coverage was 4000×–6000× in the plasma samples. The higher concordance in the relapse setting further proves that ctDNA detectability is strongly influenced by tumor activity and assay sensitivity, supporting the robustness of the cfDNA sequencing approach in the context of advanced disease.

### 3.5 Concordance between NGS and tailored ddPCR‐based approaches to detect ctDNA in EC patients

**Fig. 3:**
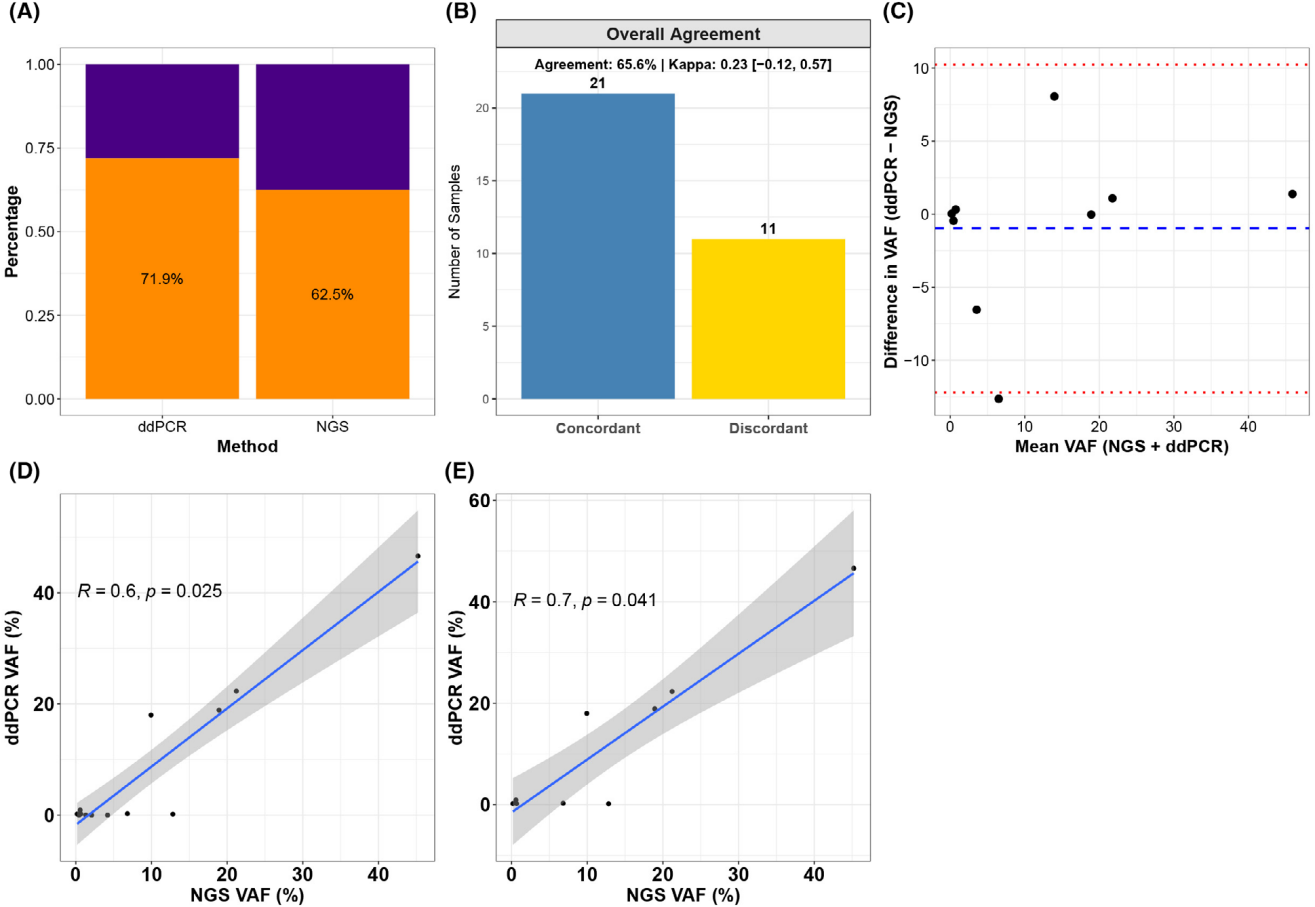
ctDNA concordance across different platforms. (A) Rate of positivity across different methodologies in all the sampling points. Orange color represents percentage of circulating tumor DNA (ctDNA) positive cases. (B) Overall agreement between platforms Kappa Cohen agreement (0.23, 95% confidence interval [−0.12, 0.57]. (C) Bland–Altman plot for the positivity rates within positive samples for both technologies. Dotted lines represent the 95% limits of agreement. (D) Spearman correlation (*R* = 0.6, *P* = 0.025) across both technologies on positive samples. Gray shaded area represents the confidence interval. (E) Spearman correlation (*R* = 0.7, *P* = 0.041) across both technologies on positive samples where the same alteration was found. Gray shaded area represents the confidence interval.

To better understand the agreement between tumor agnostic and tumor guided ctDNA assessment, in samples where both assays were run (*n* = 32), we compared the results obtained with the NGS and tailored ddPCR. Both techniques were run on the same samples to minimize sample bias. Overall, 12 patients (66.67%) presented at least one sample concordant between the ddPCR and t‐NGS approaches. Figure 3A illustrates the positivity rates across methods at a sample level; the tailored ddPCR approach showed higher positivity rates (71.88%) when compared to the t‐NGS approach (62.58%). Overall agreement between the techniques was 65.66% (Cohen's kappa: 0.23 [−0.12–0.57], Fig. 3B), indicating fair agreement. Most concordant samples were found in the context of advanced disease (*n* = 16, 50%). Regarding the VAFs, nonstatistical differences were found when comparing the results from NGS and ddPCR (Wilcoxon matched‐pairs signed rank test *P*‐value > 0.05). The Bland–Altman analysis showed a mean difference of −0.97%, with limits of agreement ranging from −12.20% to 10.26% (*n* = 9, Fig. 3C), indicating acceptable agreement between the two methods at the variant level, though with some dispersion. In cases where both methods detected pathogenic variants (*n* = 13), the VAFs correlation was moderate (Spearman, *R* = 0.6, Fig. 3D); when focusing on samples where the same mutation was detected, the correlation was higher (Spearman, *R* = 0.7, *n* = 9, Fig. 3E).

Interestingly, in Patient #ID08, tailored ddPCR detected a high *KRAS* p.G12V VAF at baseline (>45%) and a decrease at relapse (5%). However, t‐NGS did not detect *KRAS* p.G12V at baseline, even upon manual inspection using IGV (depth read 2523), whereas a 7% VAF was reported at relapse (Table S3). Conversely, in Patient #ID07, tailored ddPCR failed to detect *PIK3CA* and *FGFR2* mutations at baseline and relapse, despite their presence in t‐NGS at both time points with VAFs well above the ddPCR assay's LoD (>1%). The UA, used as a positive control, confirmed the functionality of the ddPCR probes, ruling out any technical failure.

### 3.6 Assessment of ctDNA dynamics for disease monitoring

**Fig. 4:**
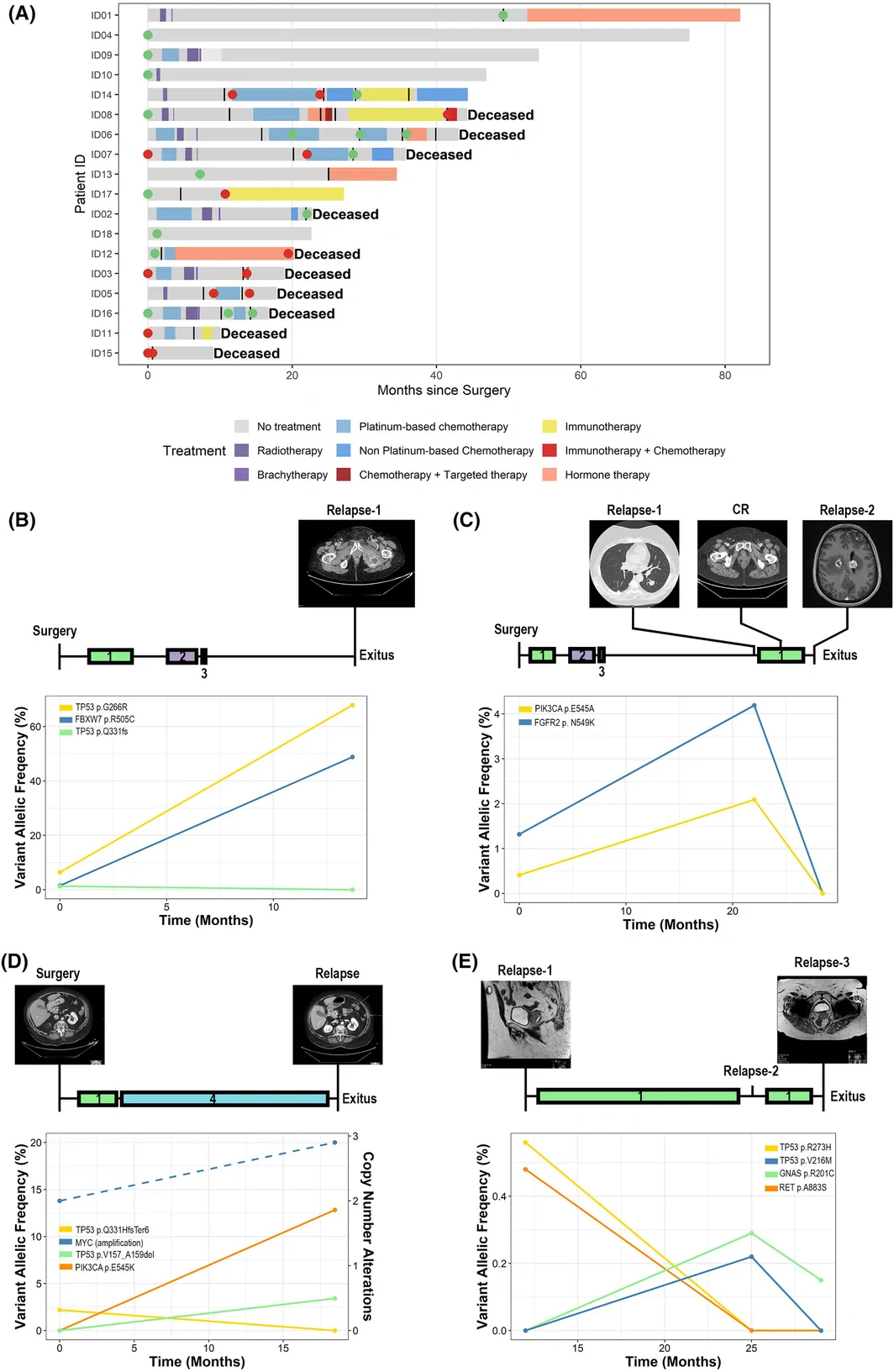
ctDNA kinetics and clinical course in high‐risk EC patients. (A) Graphical representation of longitudinal circulating tumor DNA (ctDNA) analyses in endometrial cancer (EC) patients. Circles are representative of ctDNA analyses: Green indicates noninformative samples whereas red represents samples with detectable levels of ctDNA. Black lines indicate time of relapse. Colored arms account for the different treatments patients underwent as indicated by the legend. (B–E) show ctDNA dynamics for four high‐risk EC patients with different disease stages and relapse patterns. Each panel includes ctDNA kinetics for all detectable alterations, along with computed tomography (CT)‐scan or magnetic resonance imaging (MRI) images of tumor progression and treatments. (B) Patient ID03 (C) Patient ID07 (D) Patient ID12 (E) Patient ID14. Treatments are numbered as follows: (1) Platinum based chemotherapy, (2) Radiotherapy, (3) Brachytherapy, (4) Hormone treatment.

Next, we assessed the potential applicability of ctDNA in monitoring disease evolution since one of the advantages of the NGS approach is to monitor clearance as a sign of treatment response and the opportunity to trace emerging mutations. In 8 out of 12 (66.67%) patients with plasma collected at disease relapse, we were able to detect ctDNA (at least one pathogenic variant). In 7 out of 10 (70%) patients who died due to the disease, ctDNA was detected in at least 1 sample throughout the course of the disease (Fig. 4A).

To illustrate the clinical relevance of these findings, we highlight four representative cases showcasing the value of ctDNA kinetics in tracking disease progression.

Patient #ID03 (Fig. 4B) was a 60–70‐year‐old woman diagnosed with high‐grade SC of FIGO stage IB, over 50% myometrial infiltration and no LVSI, classified as high risk for recurrence. Within a year following primary surgery, the patient experienced a relapse in the bone marrow. The UA sequencing revealed amplification of the *PI3KCA* gene and mutations in both *FBXW7* and *TP53* at surgery. Subsequent targeted sequencing of cfDNA collected at baseline and at the time of relapse identified the same mutations in *FBXW7* and *TP53*, as well as a second *TP53* alteration exclusively observed at baseline. Alterations with the higher VAF at baseline increased during relapse, indicating a higher tumor burden. Tissue immunohistochemistry (IHC) analyses confirmed an aberrant TP53 phenotype, which was concordant with the presence of *TP53* alterations in cfDNA and the UA.

Patient #ID07 (Fig. 4C) was a 50–60‐year‐old woman diagnosed with high‐grade EEC of FIGO stage IB, over 50% myometrial infiltration and LVSI, classified as high‐intermediate risk for recurrence. She developed lung metastases within 2 years after surgery and was treated with chemotherapy resulting in a complete radiological response. However, subsequently, the patient developed brain metastases. UA sequencing revealed alterations in the *PIK3CA* and *FGFR2* genes. Plasma samples were collected at the time of primary surgery, as well as first and second relapse. Alterations in *PIK3CA* and *FGFR2* were present at baseline and at the time of the first relapse. However, no detectable levels were found at the time of the second relapse. The localization to the brain may be associated with lower or nonshedding of ctDNA into the bloodstream and may therefore pose a challenge for disease monitoring by ctDNA.

Patient #ID12 (Fig. 4D) was an 80‐90‐year‐old woman diagnosed of high‐grade serous EC of FIGO stage IVA, over 50% myometrial infiltration and LVSI, classified as a patient with advanced disease. Following a chemotherapy regimen and surgical resection, the patient experienced a peritoneal relapse. UA sequencing revealed alterations in *PIK3CA*, *BRCA2*, *CREBBP*, and *PPP2R1A*. Samples were collected at the time of surgery and at the time of relapse. An alteration in *TP53* was detected in the cfDNA at baseline but not in the UA. Interestingly, this *TP53* alteration was not present in the ctDNA at the time of relapse; instead, a new *TP53* alteration emerged. Additionally, at the time of relapse, two more alterations were found: one in *PIK3CA*, concordant with the UA, and a novel amplification in *MYC*. IHC of the primary tumor showed an aberrant TP53 expression, concordant with the presence of *TP53* mutation in plasma, reinforcing the value of plasma‐based liquid biopsies to reflect intratumor heterogeneity.

Patient #ID14 (Fig. 4E) was an 80–90‐year‐old woman, diagnosed with high‐grade SC of FIGO stage IA, without any myometrial infiltration or LVSI, classified as high‐intermediate risk of recurrence. One year after primary surgery, the patient relapsed with a local recurrence. Within the subsequent year, the patient's disease progressed further. UA sequencing revealed alterations in *PIK3CA*, *RET*, and *TP53*. Samples were collected at the first, second, and third relapse. Interestingly, although alterations were detected at all three time points, the mutations identified were not consistent. Only at the time of the first relapse there were alterations concordant with tissue sequencing. Tissue IHC analyses confirmed an aberrant TP53 phenotype, which was concordant with the presence of *TP53* alterations in cfDNA and the UA.

## 4 Discussion

Liquid biopsy analyses, particularly baseline and on‐treatment ctDNA dynamics, have shown strong prognostic value in EC. Even in early stage disease, presurgical detection of ctDNA has been associated with a high risk of recurrence [14, 20, 21, 22]. In a well‐characterized cohort, predominantly composed of patients with advanced EC, we assessed the utility of an off‐the‐shelf, tumor‐agnostic t‐NGS panel. ctDNA was detectable in over 60% of samples and showed different genetic alterations than those identified in the UA in approximately 50% of patients. While the higher frequency of ctDNA and identification of unique alterations are promising, the discordances with the UA could be due to technical, methodological, or biological factors. This result underscores both the potential and the current limitations of ctDNA analysis.

The use of tumor‐agnostic methodologies offers several advantages. They allow for patient‐specific mutation tracking, monitoring of clonal evolution, and detection of actionable alterations without requiring prior sequencing of the primary tumor [21, 23, 26, 34]. Moreover, this approach may offer a more comprehensive view of the evolving mutational landscape. Applying this strategy, we were able to identify alterations in the majority of samples, in line with previously reported detection rates ranging from 20–90% depending on the technology and clinical setting [14, 21, 23, 25]. However, this approach requires relatively large cfDNA input. In our cohort, the median input for the analysis was 20.23 ng. Only one sample had input under 5ng while in most cases the remainder ranged between 16 and 60 ng. To achieve these levels, blood volumes of 10–20 mL (5–6 mL of plasma) were usually required. Over 80% of the samples achieved a LoD below 0.3%, highlighting the need for higher amounts of input material to ensure optimal analyses when moving this approach into the clinic. Fair concordance (65.66%, Cohen's kappa: 0.23 [−0.12–0.57]) and strong correlation (Spearman, *R* = 0.7) were observed when compared with a tailored ddPCR approach. Although both assays detected variants above their reported LoD, discordant results likely reflect differences in assay design or biological factors such as mutation fragment distribution. Together, these factors highlight that discordance is not unexpected and reflects both technical and biological features of cfDNA. Clinically, these discordant cases are highly relevant. It showcases that a single ctDNA modality could lead to underestimation of disease burden or missed detection of actionable alterations. Whereas tumor‐informed ddPCR approaches will fail to identify emerging clones, the tumor‐agnostic NGS may miss low‐level variants critical for early relapse detection. Our findings therefore support a complementary rather than interchangeable use of both approaches, with ddPCR being more suitable for longitudinal analyses and NGS provides broader genomic surveillance to capture clonal evolution and resistance mechanisms.

In advanced disease, longitudinal ctDNA assessment has proven highly effective to monitor the response to therapy and detect emerging resistance mechanisms, as previously reported in EC and other cancer types [25, 28, 35]. Most EC studies to date have utilized tumor‐informed strategies to assess ctDNA presence [14, 16, 20, 21, 22, 24, 26, 36]. We employed a tumor‐agnostic approach for longitudinal monitoring, as it offers a straightforward strategy that can be implemented as a single test in clinical practice. In line with previous reports across different cancer types, ctDNA was detectable in the majority of patients who experienced relapse or died from the disease, reinforcing its role as a dynamic biomarker of tumor burden [37, 38]. Our highlighted cases illustrate how ctDNA kinetics can mirror treatment response, disease progression, and the emergence of resistance mutations, supporting its utility as a minimally invasive tool to guide patient care. However, ctDNA was not detected in all patients with relapse, such as those with brain metastases, highlighting how anatomical and biological barriers may limit ctDNA shedding, leading to false‐negative results that must be carefully interpreted [39].

Notably, ctDNA analysis revealed genetic alterations not always captured in the primary tumor, including newly acquired mutations during the disease trajectory, underscoring its complementary value. Emerging alterations at relapse, such as novel *TP53* mutations or *MYC* amplifications, point to clonal evolution under therapeutic pressure and may offer novel therapeutic opportunities in advanced EC. When applying the European Society for Medical Oncology Scale for Clinical Actionability of Molecular Targets (ESCAT) [40], all identified relapse‐specific mutations fell under ESCAT level IV in endometrial tumors, indicating investigational potential with limited current clinical actionability [40]. Mutations in pathways represented in the panel such as the PI3K/AKT/mTOR pathway or CDK4/6 may guide future therapies, particularly in light of reported benefits from palbociclib–endocrine therapy combinations [7, 41, 42]. Nonetheless, pipelines using PI3K/AKT inhibitors have been stopped due to futility in the context of EC [7, 41]. Moreover, immunotherapy is increasingly used regardless of molecular subtype [7, 43], and ADCs targeting HER2 represent an additional niche where ctDNA may support therapeutic decisions [7].

A major challenge for tumor‐agnostic strategies is CH, an age‐related process where somatic mutations in hematopoietic stem cells lead to expanded clones in circulation [44, 45, 46]. As a result, plasma cfDNA may contain variants originating from blood cells rather than from the tumor, particularly in genes like *TP53*, *DNMT3A*, or *TET2* [47]. These alterations may be misattributed to the tumor, leading to false‐positive ctDNA results. In our cohort, over 50% of samples harbored at least one potential CH‐associated alteration [33, 48]. For example, given the low prevalence of *GNAS* mutations in EC (<1%), these were considered as CH‐related and the corresponding sample deemed ctDNA noninformative. Nonetheless, less than 10% of the samples within this cohort were considered as noninformative due to CH. While this strategy limited the risk of false‐positive ctDNA detection, it also highlights a key limitation of tumor‐agnostic approaches when blood‐cell–derived variants cannot be directly excluded. From a clinical implementation perspective, systematic discrimination between tumor‐derived and hematopoietic variants is essential. The most robust strategy consists of parallel sequencing of matched leukocyte DNA (buffy coat or PBMCs), enabling direct filtering of CH‐associated mutations from plasma results. Additional mitigation strategies include integration of VAF dynamics over time, prioritization of mutations with concordance to tumor tissue, exclusion of canonical CH hotspot genes, and use of error‐corrected deep sequencing to improve confidence in low‐VAF variant calls [47, 48]. However, these approaches remain limited and cannot fully substitute for leukocyte sequencing. Therefore, incorporation of matched blood controls should be considered for tumor‐agnostic liquid biopsy workflows in the clinical context.

Another caveat relates specifically to this panel's gene coverage, rather than to the use of targeted panels as a clinical strategy. While it includes many key mutations in EC, genes like *ARID1A* with high prevalence in EC tumors are absent. Besides, *PTEN* has only some sections covered, leading to potential false negatives. The restricted panel coverage underscores the critical need for caution when interpreting negative ctDNA results. A negative result cannot be conclusively interpreted as the absence of disease or actionable mutations, as it may simply reflect the lack of coverage for relevant genomic regions. Future clinical implementation should consider a broader and more EC specific panel. Larger scale panels such as Guardant360 CDx, FoundationOne Liquid CDx, or TSO500 (523 genes), as used in the IMPRESS‐Norway study (NCT04817956) [49], have shown feasibility in different clinical settings. As sequencing costs decline, such comprehensive approaches are becoming increasingly available in clinical routine. In fact, clinical trials have proven the value of including ctDNA to guide adjuvant treatments to improve the de‐escalation of treatment in various cancers [50].

## 5 Conclusions

Liquid biopsy‐based NGS is a promising tool to monitor advanced EC, detecting clinically relevant mutations and tracking disease evolution. Despite challenges such as CH and limited gene panel coverage, our approach demonstrated high mutation detection rates and concordance at relapse. These findings underscore the importance of the chosen panel's performance and sensitivity in detecting low‐frequency mutations associated with disease progression. Importantly, compared with current guidelines using tissue‐based molecular testing, plasma‐based NGS offers advantages in terms of shorter turnaround time and the potential for more cost‐effective longitudinal monitoring, particularly when repeated sampling is required. This approach shows great potential for guiding personalized treatment strategies and enhancing noninvasive disease monitoring, although further validation and optimization of the panel are necessary for clinical implementation.

## Conflict of interest

The author Kristina Lindemann reports conflict of interests outside the submitted work. KL reports grants or contracts from GSK (paid to institution); payment or honoraria for lectures, presentations, speakers' bureaus, manuscript writing, or educational events from Eisai; and participation on a data safety monitoring board or advisory board for Karyopharm, Eisai, MSD, Nykode, AstraZeneca, and GSK. The other authors report no conflicts of interest.

## Author contributions

CC‐A, KTL, ED, MA, and GM‐B: data curation, investigation, and/or formal analyses. AV, JC, EA, NG, VS, AA, and AG‐M: samples and clinical data acquisition. CC‐A, TS, KL, and LM‐R: visualization and original draft writing. EC, MA, and GM‐B: draft revision. TS, KL, and LM‐R: supervision, project administration, funding acquisition, and conceptualization. All the authors critically reviewed and approved the final draft of the manuscript. They agree to be accountable for all aspects of the work in ensuring that questions related to the accuracy or integrity of any part of the work are appropriately investigated and resolved.

## Supporting information


**Fig. S1.** Study workflow.

**Table S1.** Primary mutations identified using the Oncomine Comprehensive Panel V3 on uterine aspirates.
**Table S2.** Summary of sequencing metrics from the Oncomine Pan‐Cancer cell‐free assay.
**Table S3.** List of alterations detected in cfDNA along with associated sequencing metrics.

## Data Availability

The complete table of mutations is available in the supplementary information. The datasets generated and analyzed during the current study are available on reasonable request.
