# Supplementary figures and images for: Longitudinal circulating tumor DNA profiling in patients with advanced endometrial cancer using an off‐the‐shelf targeted NGS panel
Source: Mol Oncol. 2026 Apr 28:10.1002/1878-0261.70246. Online ahead of print. doi: 10.1002/1878-0261.70246 (PMC13398896; doi:10.1002/1878-0261.70246)

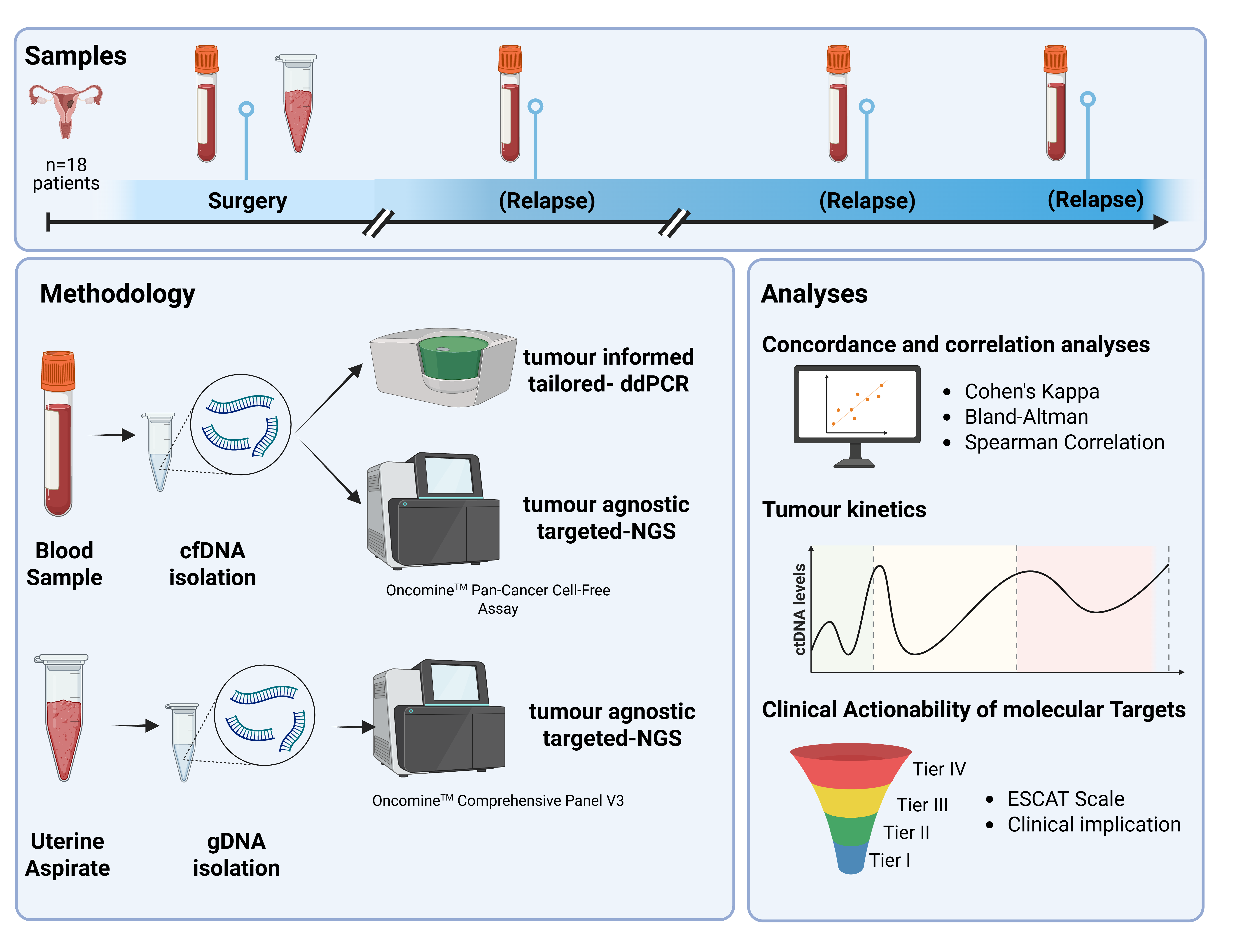

Supplement: Supplementary file 1 — Fig. S1. Study workflow. [file MOL2-9999-0-s001.png]
